# Supplementary material for: Are carbon emissions trading and green financial instruments synergistic? -Comprehensive quantitative research based on content analysis
Source: PLoS One. 2024 Mar 7;19(3):e0298601. doi: 10.1371/journal.pone.0298601 (PMC10919601; doi:10.1371/journal.pone.0298601)
Supplement: S1 Data — (ZIP) [file pone.0298601.s001.zip › Data/地级市Z_F工作报告.zip!/╔╜╢1⁄2/╠⌐░▓╩╨/└┤╫╘╣1⁄2╓┌║┼í╛┬φ┐╦╩2╛▌═°í┐_2013.docx]

各位委员、同志们：

我受政协第十二届泰安市委员会常务委员会委托，向大会作工作报告，请审议，并请列席会议的同志提出意见。

一、2012年的工作回顾

2012年是十二届市政协的开局之年，也是推进富民强市、建设幸福泰安的起步之年。一年来，在中共泰安市委的坚强领导下，市政协常委会立足新起点新使命，坚持在继承中发展、在开拓中创新，团结带领各党派团体和广大政协委员，围绕中心，服务大局，凝心聚力，促进发展，为加快推进富民强市、建设幸福泰安作出了积极贡献，人民政协事业呈现出团结和谐、蓬勃发展的良好局面。

（一）认真贯彻中央和省、市委的决策部署，坚持正确的政治方向

常委会自觉把政协工作融入全市工作大局，始终与党委思想上同心、目标上同向、工作上同步。坚持把加强学习摆在突出位置，努力建设学习型组织，通过会议部署、集中培训、专题辅导等形式，组织常委会成员和政协委员，深入学习中共十七届六中全会和十八大精神，自觉用中国特色社会主义理论统一思想、凝聚共识、引领方向；及时传达学习省委、省政协的重要会议精神，学习领会市第十次党代会和全市经济工作会议精神，准确把握全市经济社会发展的目标任务，把推进富民强市、建设幸福泰安作为政协履行职能的重点内容，研究确定了“一二三四”的工作思路，即：紧紧围绕推进富民强市、建设幸福泰安“一个中心”，牢牢把握团结、民主“两大主题”，突出抓好调研视察、提案办理和社情民意信息“三个着力点”，充分发挥委员主体、界别渠道、专委会基础、机关保障“四个作用”。围绕这个总体思路，全年安排了49项重点工作和主要活动，以奋发有为的精神状态，扎实做好维护核心、服务中心、反映民心、凝聚人心的各项工作，推动政协工作实现了新发展。

（二）紧扣中心调研议政，为加快推进富民强市进程献计出力

坚持谋大事、议全局、献良策。把开展专题调研作为协商议政的重要方式，推行自选题目和与市委商题相结合，围绕市委的重大工作部署，常委会先后开展了9项专题调研活动。围绕转变经济发展方式，组织委员对服务业发展、财源建设、外资企业发展进行调研，摸清存在问题，提出了加快发展的对策建议。围绕文化繁荣发展，吸收相关界别委员和专家组成调研组，深入基层把脉问诊，问计于民，对文化旅游服务业发展、文物保护开发利用、大汶口文化保护开发进行调研论证，分别形成了高质量的调研报告。围绕推进社会建设，抓住人民群众最关心最直接最现实的利益问题，开展了城市公共交通、企业职工维权等专题调研，为党政领导科学决策提供了重要参考。围绕建设生态文明，把泰城水源地保护和城市供水安全作为一个重要课题，深入调研，广泛论证，提出了前瞻性、可行性较强的意见建议。

这9项专题调研，是市政协全年工作的重点，凝聚了政协上下和社会各界的心血与智慧。在选题上，本着党政所需、群众所盼、政协所能的原则，抓住了富民强市中的重大问题和群众关注的热点难点问题，得到了市委市政府有关部门和基层干部群众的大力支持。在调研中，实行主席、副主席、特邀咨询分工负责，每人牵头开展一两项调研；各调研组采取实地察看、分层座谈、问卷调查、外出考察等方式，摸清情况、找准问题、探求对策。在调研报告修改过程中，广泛征求专家学者和有关部门的意见，提交政协常委会讨论，广集良策、广纳群言，形成了一批有价值有分量的建言成果，得到了市委领导的高度重视和有关方面的认真办理。市委主要领导对文化旅游业和服务业发展两个报告作出重要批示，指出“这两份报告下了很大的功夫，对情况吃得比较透，提出的意见很有价值。”市政府主要领导对政协的有关调研报告也给予高度评价，责成有关方面认真落实。同时，市政协领导同志按照市委的统一部署，包保10项重点建设项目，有力地推动了全市重点工作。

（三）认真履行民主监督职能，促进了幸福泰安建设

着眼于推进市委决策部署的贯彻落实，市政协加大民主监督力度，完善监督形式，提升监督实效，在推进富民强市、建设幸福泰安中发挥了重要作用。

完善提案办理机制，推进委员意见建议的落实。去年审查立案提案343件，经过认真研究，确定了10件重点提案、15件重要提案。为推动提案的落实，常委会认真贯彻中央、国务院和省委、省政府“两办”进一步加强人民政协提案办理工作的意见，完善提案办理运行机制，加大提案督办力度，着重从三个层面促进提案的办理。一是重点提案高层次办理。10件重点提案报市委市政府主要领导阅批，由主席副主席分工负责、各专委会协调督办，通过组织视察、座谈协商等形式，促进了提案有效落实。二是提案办理横向联动。市政协提案委与市委、市政府督查部门配合，多次召开承办部门座谈会、集体面复会、民主党派座谈会，推动提案办理落实。三是提办双方直接沟通。引导各承办单位加强与提案人联系，上门征求办理意见、面复办理情况，提升了提案落实率和委员满意率。在政协提案的推动下，城区小学建设列入市政府为民要办的实事，城乡居民基本医疗保险实现一体化统筹，泰安岩盐资源的管理和开发有了新进展，黄前水库周边环境得到进一步整治，居家养老服务体系建设加快推进，一些群众关注的热点难点问题逐步得到解决。

围绕中心搞好视察，增强民主监督实效。紧紧围绕转方式、调结构、惠民生、促发展，选择党政领导高度关注、广大群众密切关心的问题，组织开展了16项专题视察活动，深入了解情况，反映存在问题，及时向党委政府提出意见建议。紧扣主题主线，组织委员先后视察了市高新区、现代农业、质量技术监督、城市建设等工作，为加快调整经济结构出谋献策；着眼于社会管理创新，组织委员先后视察了农村医疗卫生建设、保障房建设管理、城区小学建设项目、审计法贯彻执行、人民调解、公安、人防、盐务、烟草专卖等工作，为促进社会和谐献计出力；高度关注生态文明建设，开展了林业、泰山玉矿保护开发等视察活动，推动了相关产业健康发展。市政协还与有关方面配合，完成了全国政协、省政协来我市开展的视察调研活动。

发挥信息直通车作用，提高了社情民意的时效性。常委会重视发挥社情民意信息在政协履行职能中的作用，把反映社情民意与调研视察、提案工作、委员活动结合起来，注重拓宽社情民意信息渠道，高度关注幸福泰安建设进程中的苗头性、倾向性的问题，及时反映社会管理创新中的突发性、警示性问题，为党政领导了解民情、广集民智、科学决策提供参考依据。同时通过专题辅导、加强调度、评优表彰等方式，提升了社情民意信息质量。向市委、市政府和省政协报送社情民意信息近50条，一些意见建议引起重视和采纳，产生了良好的社会效果。

强化评议监督职能，着力优化发展环境。建立了由200余名政协委员组成的评议代表备选库，按照市委统一部署，组织评议代表参加了全市政风行风情况网上投票。参评委员本着实事求是、积极审慎的态度，对91个被评议单位依法履行职责、贯彻落实政策、提升工作效能、服务全市发展等情况进行了客观公正评价，有效促进了政风行风的改善。受聘担任司法、执法等部门的53名特邀监督员，积极参加被监督部门的重要会议和活动，及时反映群众的意见，提出改进工作的建议，较好地履行了监督职责。

（四）突出发挥界别作用，为改革发展稳定凝心聚力

健全界别活动机制、发挥界别作用，是政协工作的特色和优势。一年来，市政协把加强界别建设摆在突出位置，积极探索发挥界别作用的思路和方法，依据界别和委员分布情况，把402名委员划分为27个界别小组，分别明确了组长、副组长和联络员，并召开专题会议，对界别小组工作进行动员部署；研究制定了《关于进一步发挥界别作用的意见》，明确了界别活动的制度、方式、内容和保障条件，组织开展了各具特色的界别活动。一是支持各党派团体围绕大局献计出力。政协的大会发言、专题协商、提案征集、调研视察等活动，侧重安排民主党派、工商联及其成员参加，并重视吸收和反映他们的意见。去年各民主党派市委、市工商联共提交提案67件，占集体提案总数的96%；提交政协全委会发言材料12份，占总数的57%。他们还充分发挥人才智力优势，积极开展科技培训、技术服务、健康咨询、慈善捐助、文化下乡等活动，为推进富民强市作出了积极贡献。九三学社泰安市委荣获“学习践行社会主义核心价值体系全国先进集体”。二是注重发挥民族宗教界人士的重要作用。加强与民族宗教界人士的联系，积极反映他们的诉求，维护他们的合法权益，促进了民族团结和宗教和睦。先后视察了《宗教事务条例》贯彻情况和泰城清真寺周边环境，取得了良好社会效果。三是团结港澳台侨人士助推外向型经济发展。通过“走出去、请进来”，大力宣传泰安泰山，吸引港澳台人士来泰安观光旅游、投资兴业。对全市“三胞”（港澳同胞、台湾同胞、海外侨胞）和外商投资企业信息及时进行更新，组织驻泰港澳台侨商及外资企业代表进行中秋联谊，团结他们共建幸福泰安。四是积极引导界别小组发挥作用。各界别组立足自身优势，拓宽履职渠道，开展了形式多样的活动，为促进经济社会发展作出了积极贡献。委员企业家联谊会联合有关部门，举办大型健康公益活动，为部分先模人物、环卫工人及弱势群体进行了免费体检。委员医疗保健服务团两次下乡义诊，培训医护人员250多人，为600多名村民义诊，发放了一批急需药品。文体、科教、政法等界别小组，也积极开展活动，彰显了政协的界别特色和优势。五是联合文化新闻界人士做好政协宣传和文史工作。以政协宣传和文史工作为纽带，加强相关界别人士的联系和协作，同心协力推动宣传文史工作的创新发展。市政协去年在省级以上新闻媒体发表宣传稿件120余篇，其中《人民政协报》、《联合日报》刊载近30篇。《泰安政协》获省级刊号，办刊层次和质量进一步提高。圆满完成《三峡移民工程》等资料的征集上报任务，推出了一批优秀成果。

（五）全面加强自身建设，提升了政协工作科学化水平

适应新形势新任务的要求，坚持以组织建设为重点、以作风建设为关键、以制度建设为保障，全面推进各项建设，努力提高履行职能的能力。一是着力发挥委员主体作用。围绕提升委员素质，举办了新任政协委员培训班，组织委员系统学习了政协理论和业务知识；通过印发材料、召开座谈会等形式，加强对委员的学习指导。制定了《政协委员参加活动履行职责的规定》，对委员的学习、履职、发挥主体作用、权益保障等提出明确要求，有效激发了委员履职尽责的积极性。组织委员开展了“提交一件提案，反映一条社情民意信息，开展一次专题调研，为民办一件实事”活动，在帮扶就业、捐资助学、扶贫济困、慈善救助等方面办了一批实事，产生了良好的社会影响。二是着力构建科学工作机制。把制度建设作为推动工作的一个重要抓手，围绕发挥常委会作用、规范协商议政制度、提升提案工作科学化水平等，修订完善各类制度49项，促进了政协工作制度化、规范化建设。认真落实省、市委关于加强基层政协组织建设的要求，推动各县市区建立了乡镇政协工作机构。三是着力提升机关服务效能。市政协机关开展了“创建六型机关、争作富民强市表率”活动，提素质、强能力、转作风、作表率，为政协履行职能提供了优质高效服务。创建“政协论坛”，实行机关干部轮流讲课，提升了干部的理论素养和业务能力。加强机关文化建设，努力营造团结和谐、民主求实、创新实干、敬业奉献的工作氛围，增强了干部的凝聚力、战斗力。加强了老干部工作，努力为老干部搞好服务。积极推动“强村固基”工程，全力支持驻村“第一书记”的工作，帮助基层办了许多实事好事。

各位委员，一年来，中共泰安市委认真贯彻全省政协工作会议和中共山东省委《关于加强人民政协政治协商制度建设的意见》，重视和支持政协工作，大力推进基层政协组织建设，认真吸纳政协建言成果，重视加强政协干部队伍建设，制定出台了政协提案办理意见，为全市政协事业蓬勃发展提供了坚强领导。市政府大力支持政协履行职能，定期通报全市经济社会发展情况和政协提案办理情况，密切配合政协开展调研视察活动，加大对政协提案、建议案、调研报告的办理力度，为政协履行职能创造了良好条件。政协工作所取得的成绩，是中共泰安市委正确领导的结果，是市人大、市政府和历届市政协老领导、老同志关心支持的结果，是全市各级政协组织、政协各参加单位和全体政协委员共同努力的结果。在此，我代表十二届市政协常委会，向各级党政领导和有关部门，向全市政协委员、各党派团体和各界人士，向所有关心支持政协事业发展的同志们，表示崇高的敬意和衷心的感谢！

在总结成绩的同时，也要清醒地看到，政协工作还存在一些差距和不足，主要是：政协的职能优势有待于进一步发挥；推动协商民主制度建设有待于进一步探索；履行职能的机制有待于进一步完善；机关建设水平有待于进一步提升。对这些问题，市政协常委会将认真研究解决。

二、2013年的主要任务

2013年是全面贯彻落实中共十八大精神的开局之年，也是推进富民强市、建设幸福泰安承前启后的关键一年。面对新形势新任务，我们一定要深入贯彻中共十八大和市委十届三次会议、全市经济工作会议精神，团结全市广大政协委员和各族各界人士，高举中国特色社会主义伟大旗帜，扎实履行政治协商、民主监督、参政议政职能，在推进富民强市、建设幸福泰安中，充分发挥好协调关系、汇聚力量、建言献策、服务大局的重要作用。

（一）深入学习贯彻中共十八大精神，巩固各党派团体合作共事的思想政治基础。中共十八大是在我国进入全面建成小康社会决定性阶段召开的一次重要会议。大会站在全局和战略的高度，确立了科学发展观的历史地位，确定了全面建成小康社会和全面深化改革开放的目标，对新的时代条件下推进中国特色社会主义事业作出了全面部署。同时对加强社会主义协商民主制度建设提出了新要求。党的十八大第一次将协商民主写进党代会报告，充分体现了中共中央对发展协商民主的高度重视，也赋予人民政协新的历史使命。全市各级政协组织、政协各参加单位和广大政协委员，要把学习贯彻中共十八大精神作为当前和今后一个时期的首要政治任务，在学深吃透精神实质上狠下功夫，加深理解建设中国特色社会主义“五位一体”的总布局，深刻领会全面建成小康社会的目标任务，准确把握大会对政协工作提出的新思想新论断，更加自觉地用大会精神武装头脑、凝聚共识、指导实践。通过学习，增强高举中国特色社会主义伟大旗帜、坚持走中国特色社会主义道路的坚定性，增强全面推进中国特色社会主义事业的使命感，进一步巩固全市各党派团体、各族各界人士团结奋斗的共同思想政治基础，齐心协力把建设幸福泰安的宏伟事业推向前进。

（二）发挥政协的重要渠道作用，在推进协商民主方面谋求新作为。协商民主是我国人民民主的重要形式，是实现公民有序政治参与的重要渠道。党的十八大对加强社会主义协商民主的重大意义、内容要求作了深刻阐述。人民政协作为中国共产党领导的多党合作和政治协商机构，在加强民主协商制度建设方面担负着重要任务。要充分发挥人民政协作为协商民主的重要渠道作用，完善各种协商形式，推进协商民主广泛、多层、制度化发展。要认真落实好《中共泰安市委关于加强人民政协政治协商制度建设的意见》，在协商什么、与谁协商、怎样协商、协商成果如何运用等方面深入实践，不断提高协商民主的制度化水平。要丰富协商形式，在搞好全委会议总体协商、常委会议专题协商的同时，深入推进对口协商、界别协商、提案办理协商，充分反映各党派团体、各族各界人士的意见建议。要提升协商质量，引导参与协商的各方面树立大局观念，从实际出发，认真负责地提出意见和建议，切实把协商民主这一民主形式建设好、发挥好。

（三）积极开展参政议政，为加快推进富民强市作出新贡献。要围绕市委的总体部署，把参政议政的着力点放到提高经济增长的质量和效益上，重点围绕推进大项目建设、招商引资、打造文化旅游产业高地、发展战略性新兴产业、推进城乡一体发展等关系全局的重大问题，搞好调查研究，多献务实之策。要顺应民意，围绕民生问题搞好建言献策，推动发展成果更好地惠及全市人民。要关注生态，把生态文明建设作为参政议政的重要内容，围绕加快推进绿色发展、循环发展、低碳发展，精心选择课题，深入调研论证，为建设天蓝、水绿、山青宜居家园，多建有用之言。要创业实干，全市各级政协组织、政协各参加单位和广大政协委员，要发挥好人才荟萃、智力密集、联系广泛的优势，多谋富民之策，多兴利民之举，多办惠民之事，为建设幸福泰安作出更大贡献。

（四）加大民主监督力度，为增进民众福祉发挥更大作用。要适应新形势、新任务对政协民主监督的新要求，大胆创新，突出重点，努力在民主监督上实现新突破。要认真履行民主监督职责，善于察实情、敢于讲真话，切实通过监督促进党政决策落实和群众利益的实现。要积极创新民主监督形式，探索做好提案、视察、评议等工作的新途径，推动政协民主监督与党内监督、法律监督、行政监督、舆论监督相结合，形成监督合力。要完善民主监督机制，围绕规范和加强民主监督，完善运行机制、反馈机制、激励机制、保障机制，增强监督实效。要牢固树立履职为民的思想，倾听群众呼声，体察群众疾苦，及时准确地了解和反映群众诉求，使各项政策措施更加符合群众利益。

（五）着力加强自身建设，开创政协工作新局面。要把自身建设摆在十分重要的位置，进一步促进党派合作，突出界别特色，发挥委员主体作用、专门委员会基础作用和政协机关的保障作用，更好地承担起党和人民赋予的光荣使命。要探索建立民主党派在政协开展经常性活动的机制，为他们在政协参政议政创造良好条件。要充分体现政协的界别特色，丰富界别活动形式，完善界别活动机制，努力使政协界别活动更加经常、更加规范、更加有效。要强化委员履职能力建设，完善绩效评估、督查落实等工作机制，提高委员履职成效。要切实加强专委会建设，加大创新力度，规范工作程序，使专委会的作用得到充分发挥。要加强对县市区政协的联系和指导，强化工作的协作配合与交流，进一步推进乡镇（街道）政协工作机构建设。要进一步加强政协机关作风建设，努力建设高素质的干部队伍，为政协履行职能提供优质高效的服务。

各位委员，中共十八大为人民政协进一步发挥作用指明了方向，实现富民强市新跨越为政协履行职能提供了广阔舞台。让我们更加紧密地团结起来，在中共泰安市委坚强领导下，解放思想、开拓创新、凝聚力量、团结奋进，为加快推进富民强市、建设幸福泰安，作出新的更大的贡献！
